# To explore the therapeutic effect of Kangfuxiaomi shuan II on cervicitis in rats

**DOI:** 10.1590/ACB361006

**Published:** 2021-11-29

**Authors:** Xiao-Mei Duan, Yu-Sheng Xu, Xiu-Qin Li, Xiu-Ying Ma, Jian-Ting Zhao, Gui-Ke Li, Heng Liu

**Affiliations:** 1 MM. Yunnan University of Bussiness Management – Yunnan, China.; 2 MD. Hunan Agricultural University - Hunan, China.; 3 MM. Henan Medical and Health Technician College - Henan, China.; 4 MB. Department of Pharmacy - Dali Prefecture Maternal and Child Health Care Hospital - Yunnan, China.; 5 MM. Yunnan Provincial Key Laboratory of Entomological Biopharmaceutical R&D - Dali University - Yunnan, China.; 6 MM. Jiangxi Zhong Hong Bo Yuan Biological Technology Co. Ltd - Jiangxi, China.; 7 MD. Institute of Entomoceutics Research – National Local Joint Engineering Research Center of Entomoceutics - Dali University - Yunnan, China.

**Keywords:** Periplaneta, Cervicitis, Cytokines, Rats

## Abstract

**Purpose:**

The rat cervicitis model was established with 20% phenol glue to explore the therapeutic effect of Kangfuxiaomi shuan II on rat cervicitis and its mechanism.

**Methods:**

After modeling, the rats were treated with Shuangzuotai suppository (37.84 mg/kg), Kangfuxiaoyan shuan (205.6 mg/kg) and Kangfuxiaomi shuan II (40, 80, 160 mg/kg). The histopathological changes and injury degree of cervix in rats were evaluated by vulvar inflammation score and organ index. The therapeutic effect of Kangfuxiaomi shuan II on cervicitis was evaluated by detecting the levels of copper-protein (CP), C-reactive protein (CRP), Rat interleukin 6 (IL-6), superoxide dismutase (SOD) and malondialdehyde (MDA) in serum and epidermal growth factor (EGF), cyclooxygenase-2 (COX-2) and inducible nitric oxide synthase (iNOS) in cervical tissue.

**Results:**

Compared with the model group, the vulvar inflammation score and cervical index of rats in other groups decreased significantly (*P<*0.01). Kangfuxiaomi shuan II could significantly reduce the levels of CP, CRP, and MDA in serum of rats with cervicitis, and significantly increase the activity of SOD in serum of rats with cervicitis (*P*<0.01). The levels of EGF and iNOS in cervical tissue of rats also increased in different degrees, while the level of COX-2 decreased significantly (*P*<0.01), which significantly improved the pathological degree of vulvar inflammation in rats with cervicitis.

**Conclusions:**

Kangfuxiaomi shuan II has a certain therapeutic effect on cervicitis in rats, and its mechanism may be related to the regulation of inflammatory cytokine network and immunity.

## Introduction

Cervicitis is a common clinical inflammatory syndrome. The main causes of cervicitis are cervical injury and pathogen infection, and the main pathological changes are inflammation and exudation of the columnar epithelium of the uterine endocervix^1^. Inadequate treatment of acute cervicitis often leads to chronic cervicitis^2^, which would gradually develop into cervical erosion, endometritis, or pelvic inflammation, etc., and even adjacent organ will be infected, such as cystitis^3^^,^ 4. Western medicine is often used in the clinical treatment of cervicitis. Although antimicrobials and hormones work better in the short term, the long-term use of a large number of antibiotics will result in drug resistance, disorder of self-flora, decline of immunity, etc^5^^,^ 6. Therefore, it is necessary to find an appropriate alternative treatment.

It was found that the extracts of dry worm of *Periplaneta americana* Linnaeus have the effects of anti-inflammation^7^, detumescence^8^, improving immunity^9^^,^ 10, anti-microbial^11^ and so on. Due to the good wound repair effect, the Kangxin liquid of *P. americana* Linnaeus is often used in clinical adjuvant treatment of gynecological diseases such as cervicitis and postoperative cervical erosion, which can accelerate wound healing and reduce bleeding, the risk of the secondary infection and also cervical adhesion^12^^,^ 13.

In this study, the compound extract of *P. americana* was used to prepare suppositories, named Kangfuxiaomi shuan II, which was used to treat cervicitis in rats to investigate the effect of this medicine, which laid the foundation for the follow-up development of a new traditional Chinese medicine preparation for the treatment of cervicitis.

## Methods

Sprague Dawley (SD) rats were provided by Hunan Lesk Jingda Experimental Animal Co. (7-9 weeks, female infertile, weighing 180-220 g, license number: SCXK (Xiang) 2017-0004, batch number: 20170624), and adaptive feeding of individually ventilated caging (IVC) system (22-26°C, 50% RH) in Experimental Animal Center of Dali University. All experimental animals complied with the guidelines of the China Ethics Committee on animal research.

### Instruments

- Electronic balance (Mettler Toledo, model ML204/02);
- Ultraviolet-visible spectrophotometer (Persie Analytics, model T6 New Century);
- Enzyme meter (Austria Anthos Company, model 201);
- Electric blast drying box (Beijing Everbright Medical treatment instrument Co., model 101-3E).

### Reagents

Kangfuxiaoyan shuan (Kuihua Pharmaceutical Co., Batch number 20131124); shuangzuotai shuan (Hubei Dongxin Pharmaceutical Co., Batch number 20150416); hard fat (AR, Chengdu Lutianhua Kesen Co., Batch number: 141004); kangfuxiaomi shuan II (provided by the preparation section of the Institute of Insect Biomedicine); copper-protein (CP) kit (Batch number 20170328); C-reactive protein (CRP) kit (Batch number 20170714); malondialdehyde (MDA) kit (Batch number 20170628); superoxide dismutase (SOD) kit (Batch number 20170606); inducible nitric oxide synthase (iNOS) kit (Batch number 20171014); epidermal growth factor (EGF) kit (Batch number 20170812); and cyclooxygenase-2 (COX-2) kit (Batch number 20170416) were all provided by Nanjing Jiancheng Bioengineering Institute, likewise Rat interleukin 6 (IL-6) enzyme-linked immunosorbent assay (ELISA) kit (Neobioscience Co., Batch number R170828-002a).

### Preparation of drugs

- Kangfuxiaomi shuan II: the prescription amount of mixed fatty acid glycerides was dissolved in a water bath at 60°C, and then the prescription doses of drugs, anhydrous ethanol and glycerol were stirred and mixed to prepare low, medium, and high doses of suppositories (8, 16 and 32 mg per suppository);
- Shuangzoltai suppositories and Kangfu antiphlogistic shuan: taking appropriate amount of Shuangzuotai shuan and Kangfu anti-inflammatory suppositories sold in the market, add mixed fatty acid glycerides in a water bath at 60°C, stir and mix well, and prepare each suppository containing 7.57 and 41.12 mg, respectively;
- 20% phenol glue: liquid phenol 4 mL and 1 g sodium carboxymethyl cellulose were mixed with 6 mL glycerol, then diluted with 9 mL distilled water and stored in a sealed refrigerator at 4°C (mixed at room temperature during the experiment).

### Establishment of the model

To establish the model of cervicitis in rats by phenol glue. Healthy female non-pregnant rats aged 7-9 weeks were selected, of which 10 were randomly selected as normal control group, and the remaining rats were used to make cervicitis model. The rats were anesthetized with isoflurane inhalation for 30 s and then fixed. The syringe was gently inserted into the cervical dome of the rat with a sterilized syringe of 1 mL size, and 20% phenol glue was slowly pushed into the cervix. Then, the rats were placed in the cage after 5 min, according to the requirement of high head and low tail. The model was made once a day in the first three days, and each animal was 0.15 mL. The model was made again after an interval of one day, and each animal was 0.2 mL. The model was made once a day for the next three days, and each animal was 0.15 mL^14^^,^ 15. The normal group was operated in the above way, in which the modeling reagent was replaced by normal saline.

### Grouping and model of administration

Three days after the last modeling, the changes of peripheral vaginal inflammation in rats were observed with naked eyes. According to the score standard of vulvar inflammation^16^ (Tables 1 and 2), the rats were divided into four grades: no inflammation, mild inflammation, moderate inflammation, and severe inflammation. The rats without inflammation (five rats) and with severe inflammation (six rats) were excluded, and the remaining model rats were randomly divided into seven groups, according to the order of mild inflammation (38 rats) and moderate inflammation (32 rats): model group, Kangfuxiaomi shuan II vehicle group (vehicle group), low-, middle- and high-dose groups of Kangfuxiaomi shuan II (PA-II-L, PA-II-M, PA-II-H), Shuangzuotai shuan group (SZTS group) and Kangfuxiaoyan shuan group (KFXYS), (n = 10).

**Table 1:** The indexes of vulvar inflammation in rats with cervicitis induced by phenol glue.

Symptoms	Score	Symptoms	Score	Symptoms	Score
No erythema	0	No edema	0	No secretion	0
Very mild erythema	1	Very mild edema	1	Very few secretions	1
Mild erythema (dim red)	2	Mild edema	2	Few secretions	2
Moderate erythema(obvious red)	3	Severe edema (protruding about 1 mm)	3	Moderate secretion	3
Severe erythema (fuchsia)	4	Severity edema (bulge greater than 1 mm)	4	A large amount of secretions	4

**Table 2:** Grading criteria of vulvar inflammation in rats with cervicitis induced by phenol glue.

Score	Grading	Score	Grading
0-3	Normal (no inflammation)	7-9	Moderate inflammation
4-6	Mild inflammation	10-12	Severe inflammation

Normal group and model group were given normal saline at the dose of 0.05 mL/100 g. The low-, middle- and high-dose groups of Kangfuxiaomi shuan II (40, 80, 160 mg/kg), Shuangzuotai shuan group (37.84 mg/kg), Kangfuxiaoyan shuan group (205.6 mg/kg) and model group (0.2 g vehicle) were given intravaginally for 10 days. The erythema, edema, and secretion of vaginal mouth of rats in each group were observed and scored on the 1st, 6th, and 11th day after administration.

Fasting could not abstain from water for 24 h after the last administration. The rats were anesthetized by intraperitoneal injection of 10% chloral hydrate solution (3 mL/kg), and the rats were euthanized by injection of excessive anesthetic after blood extraction from abdominal aorta. The blood was taken from the abdominal aorta and placed at 4°C for 4 h. The upper serum was obtained by 3,000 r/min centrifugation for 10 min. The expression levels of CP, CRP, IL-6 and the activities of MDA and SOD were detected half of the cervical tissues were taken and the levels of EGF, COX-2 and iNOS were detected by ELISA. Kidney and cervix were weighed and calculated to get the organ index (organ index = organ weight/body weight×100%). The residual cervical tissue of rats was fixed with 40 mg/mL formaldehyde to make pathological sections and histopathological score (HS)^17^^,^ 18. The pathological changes and grading criteria of cervical tissue were graded according to Tables 3 and 4.

**Table 3:** Grading and evaluation of histopathological changes of cervix in rats.

Histopathological changes (+)	Histopathological changes (++)	Histopathological changes (+++)
Local thickening of 2-3 layers of squamous epithelium	Significant thickening of squamous epithelium	Extensive thickening and uneven mucous membrane
A small amount of cell edema in squamous epithelium	Obvious edema of squamous epithelium	Massive cell edema
The columnar epithelium has a squamous tendency	Obvious squamous metaplasia of columnar epithelium	The squamous area is widely used to reach the uterine cavity
A small amount of infiltration in the mucosa and sublayer was slightly more than that in the control group	Increased mucosal and submucosal interstitial infiltration	Massive infiltration and proliferation in the deep layer of mucous membrane and stroma, accompanied by hyperemia
Only fibroblasts in the stroma were slightly more fertile than those in the normal control group	Fibroblasts grow and have a small number of collagen fiber bundles	Fibroblast proliferation is accompanied by the formation of a large number of collagen bundles
Only a small amount of telangiectasia and hyperemia and interstitial edema were seen in the submucosa	Hyperemia and edema can be seen in the submucosa	A large number of hyperemia and edema can be seen in the submucosa

**Table 4:** Grading and evaluation of histopathological changes of cervix in rats.

Grading	Degree of inflammatory changes
Normal	Less than two items of histopathological changes (+)
Mild	More than two items of histopathological changes (+)
Moderate	More than two items of histopathological changes (+), and two items of histopathological changes (++); or one item of histopathological changes (++) and more than three items of histopathological changes (+++)
Severe	One item of histopathological changes (+++) and two items of histopathological changes (++) and two items of histopathological changes (+); or two items of histopathological changes (+++) and one item of histopathological changes (++) and one item of histopathological changes (+); or three items of histopathological changes (+++)

### Data processing

The software Statistical Package for the Social Sciences (SPSS) 24.0 was used to carry out the test. Each group of experimental data is represented by *x*±*s*, the data in accordance with the normal distribution are tested by variance test, and the data that do not conform to the normal distribution are tested by rank-sum test. There was a statistical difference in terms of *P<*0.05 or *P<*0.01.

## Results

### Effect of PA-II on the general state of cervicitis in rats

The hair of the rats in the normal group was smooth, and the activity was normal. After the establishment of the cervicitis model, the rats were in bad mental state and irritable to gather, erect and had dull hair. On the 6th day after administration, the mental state of rats in the middle- and high-dose groups of Kangfuxiaomi shuan II, Shuangzuotai shuan group and Kangfuxiaoyan shuan group was significantly improved, and their diet and water drinking were normal. The mental state of rats in the Shuangzuotai shuan group was worse than that in the Kangfuxiaoyan shuan group. On the 11th day of administration, rats in Kangfuxiaoyan shuan group, middle- and high-dose group of Kangfuxiaomi shuan II, and mental state of rats in Shuangzuotai shuan group gradually improved with slight clustering phenomenon.

### Effect of PA-II on vulvar inflammation in rats

Compared with the normal group, the vulvar inflammation scores of rats in model group, Shuangzuotai shuan group, Kangfuxiaoyan shuan group, and Kangfuxiaomi shuan II group were significantly higher than those in normal group (*P<*0.01).

On the 6th day after administration, the erythema and edema of vaginal orifice of rats in each group gradually disappeared. Compared with the vehicle group, the vulva score of rats in the middle- and high-dose groups of Kangfuxiaomi shuan II decreased significantly (*P<*0.01). On the 11th day after administration, the erythema of vaginal orifice disappeared, the vaginal orifice of some rats showed slight edema and secretion decreased.

Compared with the model group and vehicle group, the score of vulvar inflammation in each dose group of Kangfuxiaomi shuan II was significantly lower than that in the model group and vehicle group (*P<*0.01). Compared with model group and vehicle group, the score of vulvar inflammation in Shuangzuotai shuan group and Kangfuxiaoyan shuan group was significantly lower than that in model group and vehicle group (*P<*0.01) (Table 5).

**Table 5:** Effect of PA-II on vulvar inflammation in rats (*x*±*s*, n=10) § .

Group	Dose(mg/kg)	Day 1	Day 6	Day 11
Normal	---	0.0±0.00	0.0±0.00	0.0±0.00
Model	---	6.4±1.2 **	6.6±0.5	6.7±0.8
Vehicle	---	6.6±0.5 **	6.9±1.2	6.8±0.7
SZTS	37.84	6.6±0.7 **	5.1±1.2 ## ΔΔ	5.1±1.5 ## ΔΔ
KFXYS	205.6	6.4±0.5 **	4.6±1.1 ## ΔΔ	4.4±1.1 ## ΔΔ
PA-II-L	40	6.4±0.8 **	5.9±0.9 ∇	4.5±1.3 ## ΔΔ
PA-II-M	80	6.4±0.5 **	5.6±1.3 Δ	4.6±1.2 ## ΔΔ
PA-II-H	160	6.6±0.5 **	5.5±1.3 Δ	4.4±1.6 ## ΔΔ

§ Data are presented as mean ± standard deviation of 10 rats in each group: *P < 0.05,

** P < 0.01 *vs*. normal group; ^#^P < 0.05;

## P < 0.01 *vs*. model group;

∇ P < 0.05, ∇∇P < 0.01 *vs*. vehicle group;

Δ P < 0.05,

ΔΔ P < 0.01 *vs*. KFXYS group.

### Effect of PA-II on organ index of cervicitis in rats

**Figure 1:**
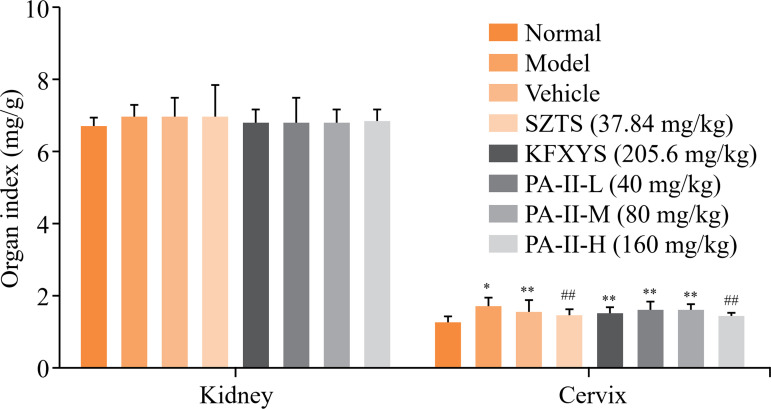
Effect of PA-II on organ index of cervicitis in rats. Data are presented as mean ± standard deviation of 10 rats in each group.

Compared with the normal group, the renal index of rats in each model group increased in varying degrees, but there was no statistical difference. After being treated with Kangfuxiaomi shuan II, the cervical index of rats in Kangfuxiaomi shuan II high-dose group and Shuangzuotai shuan group decreased significantly (*P<*0.0l) (Fig. 1).

*P < 0.05; **P < 0.01 *vs*. Normal group; ^#^P < 0.05; ^##^P < 0.01 *vs*. model group.

### Effects of PA-II on serum CP, IL-6, CRP, MDA and SOD in rats with cervicitis

**Figure 2:**
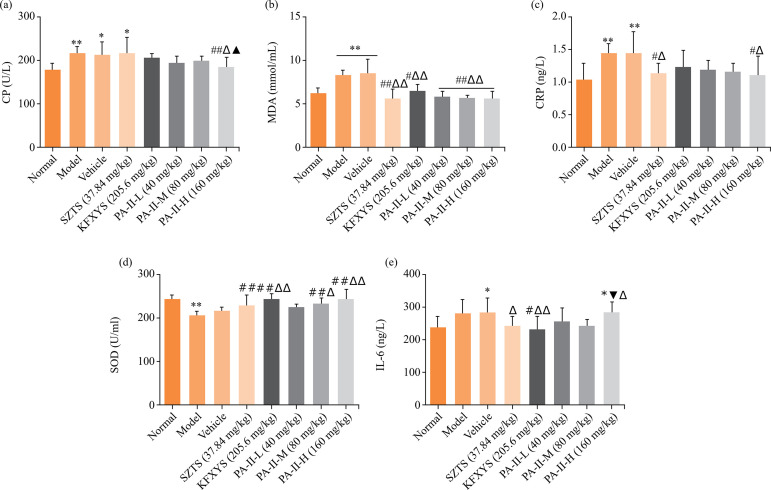
Effects of PA-II on serum CP, IL-6, CRP, MDA and SOD in rats with cervicitis. Data are presented as mean ± standard deviation of 10 rats in each group.

CP: copper-protein; CRP: C-reactive protein; IL-6: rat interleukin 6; MDA: malondialdehyde; SOD: superoxide dismutase; *P < 0.05; **P < 0.01 vs. normal group; ^#^P < 0.05; ^##^P < 0.01 vs. model group; ^∆^P < 0.05; ^∆∆^P < 0.01 vs. vehicle group; ^▲^P < 0.05; ^▲▲^P < 0.01 vs. SZTS; ^∇^P < 0.05; ^∇∇^P < 0.01 vs. KFXYS group.

Compared with the normal group, the activities of CP, IL-6, CRP, and MDA in serum of rats in each model group increased in different degrees. After treatment with Kangfuxiaomi shuan II, the activities of CP, IL-6, CRP, and MDA in serum of rats in the model group were significantly improved as compared with those in the model group (Fig. 2 a-d). Compared with the normal group, the level of serum SOD in the model group was significantly lower than that in the normal group (*P<*0.01). Compared with the model group, the level of SOD in serum of rats in middle- and high-dose groups of Kangfuxiaomi shuan II, Shuangzuotai shuan and Kangfuxiaoyan shuan increased significantly (*P<*0.01) (Fig. 2e).

### Effect of PA-II on EGF, COX-2 and iNOS in cervical tissue of rats with cervicitis

**Figure 3:**
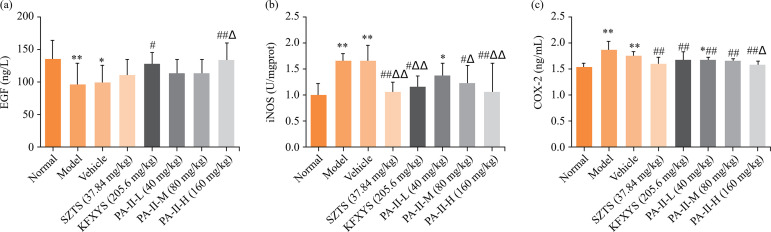
Effects of PA-II on serum CP, IL-6, CRP, MDA and SOD in rats with cervicitis. Data are presented as mean ± standard deviation of 10 rats in each group.

EGF: epidermal growth factor; iNOS: inducible nitric oxide synthase; COX-2: cyclooxygenase-2; *P < 0.05; **P < 0.01 vs. normal group; ^#^P < 0.05; ^##^P < 0.01 vs. model group; ^∆^P < 0.05; ^∆∆^P < 0.01 vs. vehicle group.

Compared with the normal group, the content of EGF in cervical tissue of rats in each model group decreased significantly (*P<*0.01). The content of EGF in cervical tissue of rats in high-dose group of Kangfuxiaomi shuan II and Kangfuxiaoyan shuan increased after administration (*P<*0.05 or *P<*0.01) (Fig. 3a). Compared with the normal group, the levels of COX-2 and iNOS in the cervical tissue of rats in each model group were higher than those in the normal group (*P<*0.01). The levels of COX-2 and iNOS in cervical tissue of rats in each treatment group were improved in varying degrees after treatment (Fig. 3 b-c).

### Effect of PA-II on cervical histopathological score in rats with cervicitis

**Figure 4:**
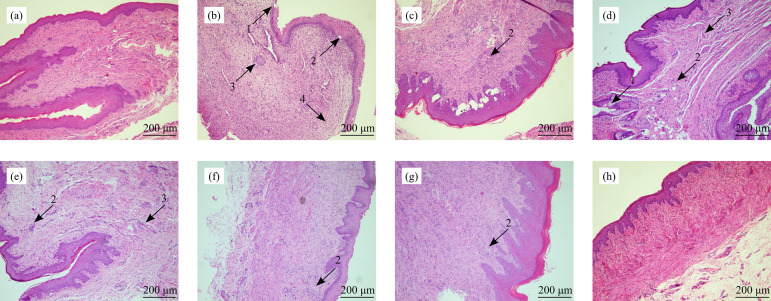
Effect of PA-II on cervical histopathological images in rats with cervicitis (scale bar: 200 μm). **(a)** Cervical tissue sections of rats in the normal group. **(b)** Cervical tissue sections of rats in the model group. **(c)** Cervical tissue sections of rats in vehicle group. **(d)** Cervical tissue sections of rats in the SZTS group. **(e)** Cervical tissue sections of rats in the KFXYS group. **(f)** Cervical tissue sections of rats in the low-dose group of PA-II. **(g)** Cervical tissue sections of rats in the middle-dose group of PA-II. **(h)** Cervical tissue sections of rats in the high-dose group of PA-II.

The HE section of cervical tissue was observed by medical image analysis system. Slight squamous epithelium thickening, slight edema, no obvious inflammatory cell infiltration, and fibroblast proliferation were found in the cervical tissue of rats in the normal group (Fig. 4a). Compared with the normal group, the cervical tissue in the model group showed thickening of squamous epithelium, edema, a large number of inflammatory cells infiltrating into the submucosa, and fibroblast proliferation (Fig. 4b). Compared with the model group, the inflammatory cell infiltration area of cervical tissue in Shuangzuotai shuan and Kangfuxiaoyan shuan decreased, fibroblasts proliferated slightly, likewise edema (Fig. 4 d-e). In the three dose groups of Kangfuxiaomi shuan II, the area of inflammatory cell infiltration in the cervical tissue decreased, the capillaries were slightly congested, and there was no edema (Fig. 4 f-h).

1: Loss of squamous epithelium; 2: inflammatory cell infiltration; 3: fibroblast proliferation; 4: edema.

**Figure 5:**
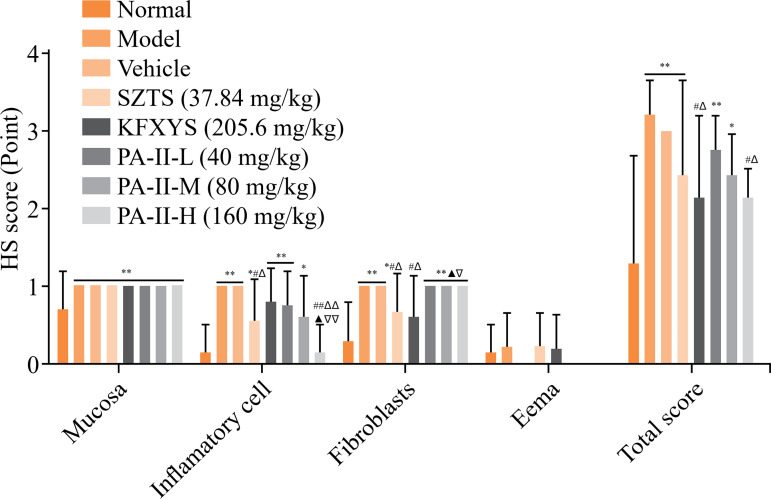
Effect of PA-II on cervical histopathological score in rats with cervicitis. Data are presented as mean ± standard deviation of 10 rats in each group.

Compared with the normal group, the scores of fibroblast proliferation, inflammatory cells, and HS in the cervical tissue of the model group were significantly higher than those of the normal group (*P<*0.01). Compared with the model group, the HE section, and scores of inflammatory cell infiltration in the middle-dose group and Shuangzuotai shuan group of Kangfuxiaomi shuan II were significantly lower than those in the model group (*P<*0.05 or *P<*0.01). Compared with the model group, the total score of cervical histopathological indexes in other groups decreased, and the total score of Kangfuxiaomi shuan II medium-dose group decreased significantly (Fig. 5).

*P < 0.05; **P < 0.01 *vs*. normal group; ^#^P < 0.05; ^##^P < 0.01 *vs*. model group; ^∆^P < 0.05; ^∆∆^P < 0.01 *vs*. vehicle group; ^▲^P < 0.05; ^▲▲^P < 0.01 *vs*. SZTS; ∇P < 0.05; ∇∇P < 0.01 *vs*. KFXYS group.

## Discussion

Phenol is highly corrosive as a chemical reagent to the skin and mucous membrane, and it will damage the vaginal mucosa and cause inflammation after vaginal contact. In this experiment, the rat cervicitis model was established by 20% phenol glue belonging to mild to moderate inflammation, which has a certain effect on the cervical tissue of rats. After the establishment of the cervicitis model, light red fine particles appeared in the cervix and vaginal orifice of rats. The cervical tissue of rats showed obvious thickening of squamous epithelium and mucosal layer, edema, and a large number of inflammatory cells infiltrated into the submucosa. That was in line with the pathological characteristics of clinical acute cervicitis. The success rate of the model was high, and the experimental cycle was short.

Kangfuxiaoyan shuan is a compound preparation composed of a variety of traditional Chinese medicine ingredients, which has a certain clinical effect in the treatment of cervicitis. However, the effect of reducing cervical index in this experiment is weak, which may be due to the complex composition of this medicine. The absorption of animals has a certain effect on their own immune organs and urinary system. After treated with Kangfuxiaomi shuan II, the cervical index of rats decreased.

In the pathophysiological mechanism of the occurrence and development of cervicitis, the release of a variety of inflammatory factors has become an important risk factor in the development of cervicitis^19^. CP and CRP in the model group increased significantly. After treatment with Kangfuxiaomi shuan II, the expression of CP and CRP was inhibited, and the inflammation was relieved. IL-6 is a cytokine secreted by lymphocytes and plays an important role in cellular immune regulation, hematopoiesis and inflammation^20^. NO is an important regulator of information transmission between cells. When inflammation occurs, it can induce the synthesis of iNOS. When the body is infected, iNOS and COX-2 will be produced in large quantities. In this experimental model group, the levels of COX-2 and iNOS were significantly increased, and the levels of COX-2 and iNOS decreased in varying degrees after treatment with Kangfuxiaomi shuan II. The high-dose group of Kangfuxiaomi shuan II could significantly inhibit the expression of iNOS, relieve edema and reduce the score of vulvar inflammation in model rats. SOD activity represents the ability to resist free radical damage, and the amount of MDA content reflects the degree of inflammation of the body^21^. Each group of Kangfuxiaomi shuan II could significantly increase the expression of SOD, inhibit the production of MDA, and reduce oxidative damage.

## Conclusion

Kangfuxiaomi shuan II can effectively repair cervical tissue injury in rats, improve the ability of antioxidation, enhance the ability of tissue repair, reduce tissue hyperemia and edema, and inhibit the occurrence of inflammation. So, it has a certain therapeutic effect on rat cervicitis, and provide experimental basis for the treatment of clinical cervicitis.
